# Adiposity markers and insulin resistance in type 1 diabetes patients

**DOI:** 10.1186/1758-5996-7-S1-A101

**Published:** 2015-11-11

**Authors:** Camila Lemos Marques, Jussara Carnevale Almeida, Ticiana da Costa Rodrigues

**Affiliations:** 1 UFRGS, Porto Alegre, Brazil

## Background

Obesity is associated with risk of clinical and metabolic complications such as dyslipidemia, hypertension and diabetes. Previous studies have demonstrated that visceral obesity is related to type 2 diabetes, cardiovascular disease and insulin resistance (IR). This study aims to evaluate the relationship between different markers of body adiposity and IR in adults with type 1 diabetes mellitus (T1D).

## Materials and methods

Cross-sectional study in outpatient adults with T1D in a hospital in southern Brazil, data collected from 2008 to 2013. The anthropometric measurements used were waist circumference (WC), waist-to-height ratio (WHtR), body mass index (BMI), conicity index (CI), lipid accumulation product (LAP) and body fat index (BFI). IR was measured using estimated glucose disposal rate (EGDR) by the formula: EGDR (mg.kg-1.min-1)=24,31 – 12,22 (waist-hip ratio) – 3,29 (hypertension presence) – 0,57 (glycated hemoglobin) and was analyzed in tertiles (tertile 1 ≤ 5.4; tertile 2 between 5.4 and 8.4 and tertile 3 ≥ 8.4 mg.kg-1.min-1). Results: The 128 subjects studied (51.7% women) had mean age of 38.7±11.3 yrs. and median of EGDR 7.2 (4.4–8.7) mg.kg-1.min-1. Individuals of the first tertile of EGDR (more resistant) had higher values of WC, WHtR, CI, LAP and BMI when compared with individuals of the others tertiles. EGDR was negatively correlated with WC (r=-0.36, p< 0.01), WHtR (r=-0.39, p< 0.01), CI (r=-0.44, p< 0.01), LAP (r=-0.41, p< 0.01) and BMI (r=-0.24, p< 0.01). After gamma regression analyses, adjusted for age and serum triglycerides, WC (Beta=-0.01; p<0.01), WHtR (Beta=-2.32; p<0.01), CI (Beta=-1.91; p=0.018) and LAP (Beta=-0.007; p<0.01) remained negatively associated with EGDR.

## Conclusions

The studied adiposity markers were associated with IR, with exception of BFI (p=0.36). Markers that include WC appear to be better predictor of IR than isolated WC measure. CI, WHtR and LAP showed greater tendency to identify IR in adults with T1D. This study suggests that through anthropometric measurements is possible predict IR in adults with T1D and these measurements must be used by health professionals, since they are easily used on clinical application.

